# Pyrophosphate inhibits gluconeogenesis by restricting UDP-glucose formation *in vivo*

**DOI:** 10.1038/s41598-018-32894-1

**Published:** 2018-10-02

**Authors:** Ali Ferjani, Kensuke Kawade, Mariko Asaoka, Akira Oikawa, Takashi Okada, Atsushi Mochizuki, Masayoshi Maeshima, Masami Yokota Hirai, Kazuki Saito, Hirokazu Tsukaya

**Affiliations:** 1 Department of Biology, Tokyo Gakugei University, Koganei, Tokyo 184-8501 Japan; 2 Okazaki Institute for Integrative Bioscience, Okazaki, Aichi 444-8787 Japan; 3 National Institute for Basic Biology, Okazaki, Aichi 444-8585 Japan; 4 Department of Basic Biology, School of Life Science, Graduate University for Advanced Studies, Okazaki, Aichi 444-8585 Japan; 5 RIKEN Center for Sustainable Resource Science, Yokohama, 230-0045 Japan; 6 Laboratory of Cell Dynamics, Graduate School of Bioagricultural Sciences, Nagoya University, Nagoya, 464-8601 Japan; 7 Faculty of Agriculture, Yamagata University, Tsuruoka, 997-8555 Japan; 8 Theoretical Biology Laboratory, RIKEN, Wako, 351-0198 Japan; 9 Graduate School of Pharmaceutical Sciences, Chiba University, Chiba, 263-8522 Japan; 10 Department of Biological Sciences, Graduate School of Science, The University of Tokyo, Tokyo, 113-0033 Japan; 11 Present Address: Exploratory Research Center on Life and Living Systems (ExCELLS), Okazaki, Aichi 444-8787 Japan; 12 Present Address: Department of Biology, Tokyo Gakugei University, Nukui-Kita 4-1-1, Koganei, Tokyo 184-8501 Japan

**Keywords:** Sugar phosphates, Metabolomics

## Abstract

Pyrophosphate (PPi) is produced by anabolic reactions and serves as an energy donor in the cytosol of plant cells; however, its accumulation to toxic levels disrupts several common biosynthetic pathways and is lethal. Before acquiring photosynthetic capacity, young seedlings must endure a short but critical heterotrophic period, during which they are nourished solely by sugar produced from seed reserves by the anabolic process of gluconeogenesis. Previously, we reported that excess PPi in H^+^-PPase-knockout *fugu5* mutants of *Arabidopsis thaliana* severely compromised gluconeogenesis. However, the precise metabolic target of PPi inhibition *in vivo* remained elusive. Here, CE-TOF MS analyses of major metabolites characteristic of gluconeogenesis from seed lipids showed that the Glc6P;Fru6P level significantly increased and that Glc1P level was consistently somewhat higher in *fugu5* compared to wild type. In contrast, the UDP-Glc level decreased significantly in the mutants. Importantly, specific removal of PPi in *fugu5*, and thus in *AVP1*_*pro*_*:IPP1* transgenic lines, restored the Glc1P and the Glc6P;Fru6P levels, increased the UDP-Glc level ~2.0-fold, and subsequently increased sucrose synthesis. Given the reversible nature of the Glc1P/UDP-Glc reaction, our results indicate that UGP-Glc pyrophosphorylase is the major target when excess PPi exerts inhibitory effects *in vivo*. To validate our findings, we analyzed metabolite responses using a mathematical theory called structural sensitivity analysis (SSA), in which the responses of concentrations in reaction systems to perturbations in enzyme activity are determined from the structure of the network alone. A comparison of our experimental data with the results of pure structural theory predicted the existence of unknown reactions as the necessary condition for the above metabolic profiles, and confirmed the above results. Our data support the notion that H^+^-PPase plays a pivotal role in cytosolic PPi homeostasis in plant cells. We propose that the combination of metabolomics and SSA is powerful when seeking to identify and predict metabolic targets in living cells.

## Introduction

Inorganic pyrophosphate (PPi) was discovered in the 19^th^ century and, in 1941, was found to accumulate in rat livers; this was the first report on PPi accumulation in a biological system^1^. Later, Kornberg described the first PPi-producing biological reaction^2^ and proposed that pyrophosphorylases acted in the direction of PPi production favoring the formation of biochemical compounds^3^. Further, it was suggested that inorganic pyrophosphatase (PPase)-mediated PPi hydrolysis rendered the above reactions practically irreversible^4^, a hypothesis that is now widely accepted. PPi hydrolysis has a ∆G^′°^ of −33.4 kJ/mol and can therefore drive reactions that are otherwise energetically unfavorable, including many biosynthetic steps^5^. Almost 200 different reactions produce PPi^6–9^. The loss of PPase activity arrested growth in bacteria^10^ and yeast^11^ and triggered developmental blockage at an early larval stage in worms^12^, supporting a vital role for PPi homeostasis in living cells. In the model plant *Arabidopsis thaliana* (hereinafter, Arabidopsis), we previously reported that vacuolar proton pyrophosphatase (H^+^-PPase) is essential for maintaining adequate PPi levels^13^, and that cytosolic PPa isozymes that exhibit non-overlapping subcellular localization patterns^14^, particularly PPa1, act cooperatively with H^+^-PPase to prevent an increase in PPi concentrations to toxic levels^15^. The PPi concentration in the cytosol of plant cells was 0.2–0.3 mM^16^. Moreover, the constitutive expression of vacuolar proton pyrophosphatase (H^+^-PPase) increases plant growth under a variety of abiotic stresses, rendering the encoding gene of critical importance to crop breeders^17,18^. However, the actual target of excess PPi *in vivo* and the physiological roles of PPases remain enigmatic in all living organisms, and little is known about the master regulator of cytosolic PPi homeostasis in plants^5^.

Against this background, we isolated^19^ and characterized vacuolar H^+^-PPase loss-of-function *fugu5* mutants of Arabidopsis; these are viable but exhibit defects in cotyledon development and hypocotyl elongation^13^. The postgerminative growth defects recover when sucrose (Suc) is supplied or when PPi is removed by the yeast cytosolic PPase IPP1 in the *AVP1*_*pro*_*:IPP1* lines^13^. This indicated that H^+^-PPase played a major role in the hydrolysis of inhibitory PPi^9,13,20,21^. The PPi level was ~2.5-fold higher, and the Suc level 50% lower, in *fugu5* etiolated seedlings compared to those of wild-type (WT)^13^. Thus, excess PPi likely inhibits gluconeogenesis from seed storage lipids (triacylglycerols; TAG), but the precise metabolic target remained unclear^9,13^. Thus, we examined how excess PPi inhibited gluconeogenesis *in vivo*.

**Figure 1:**
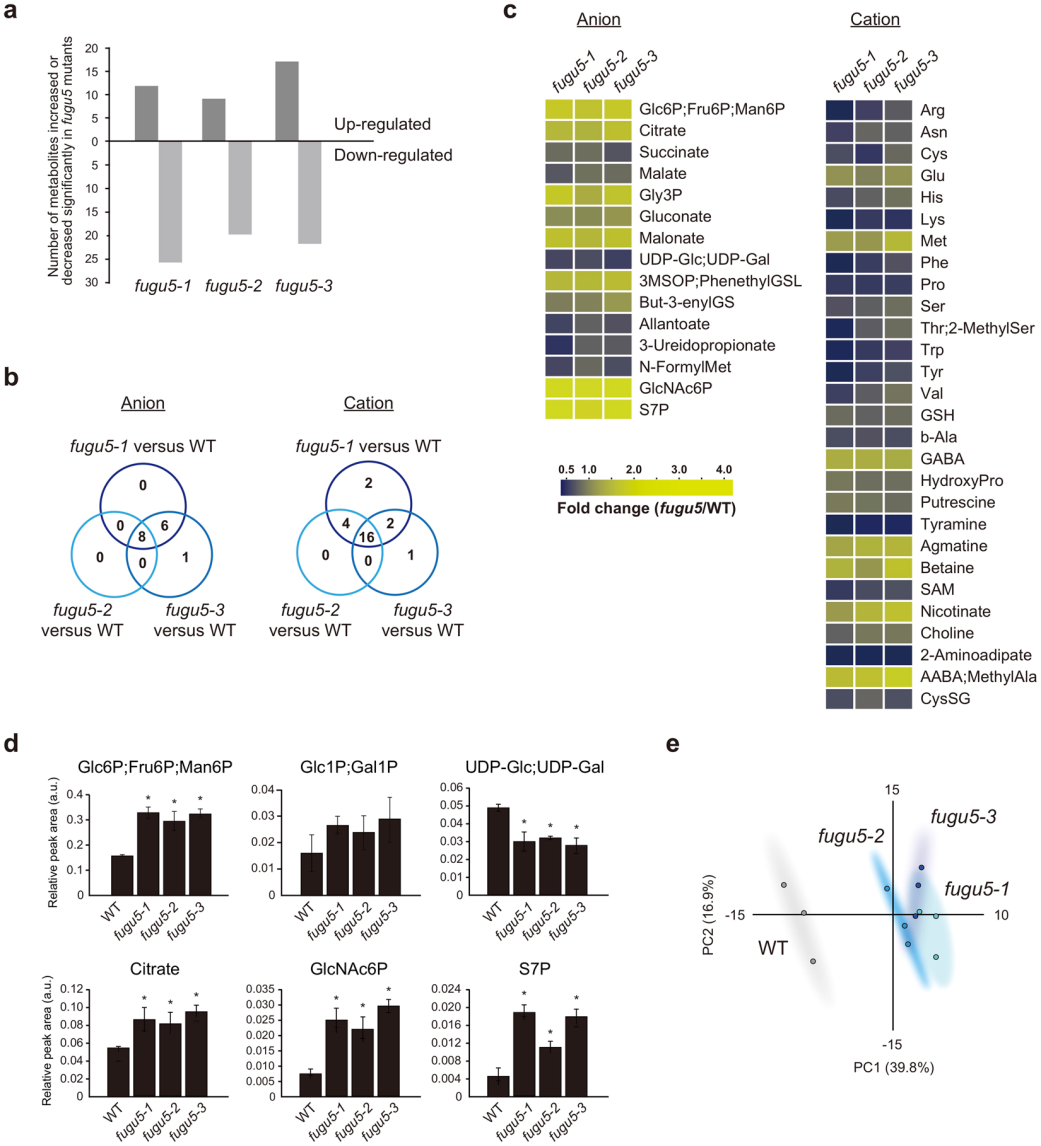
The metabolomics of *fugu5* mutants and the WT with a focus on major metabolites associated with mobilization of seed storage lipids. (**a**) The numbers of metabolites, the levels of which were significantly affected (increased or decreased) in seedlings of *fugu5* mutants compared with the WT grown in the dark for 3 days. Metabolites were analyzed with the aid of CE-TOF MS (*n* = 3). (**b**) Venn diagrams showing the numbers of overlapping metabolites in the WT and *fugu5* mutants. (**c**) Comparison of the metabolite levels in the *fugu5* and WT strains. Metabolites, the levels of which increased or decreased significantly as revealed by the two-tailed Student’s *t*-test (*P* < 0.05, *n* = 3), are color-coded, and the fold changes in the *fugu5* strains compared with the WT are shown. Left: anions; right: cations. Metabolites that are not resolved by CE-TOF MS (Glc6P, Fru6P, and Man6P; Glc1P and Gal1P; and UDP-Glc and UDP-Gal) are shown together. (**d**) Statistical analysis of the normalized datasets of key metabolites that were significantly affected. All data are means ± SDs (*n* = 3). Asterisks indicate significant differences compared to the WT (*P* < 0.05, two-tailed Student’s *t*-test). (**e**) The score plot yielded by principal component analysis (PCA). Abbreviations are summarized in Supplementary Table S3.

Here, CE-TOF MS analyses of major metabolites produced during TAG mobilization showed that relatively few metabolites were significantly affected in three *fugu5* alleles compared to WT (Fig. 1a). In fact, only 8 anions and 16 cations were commonly up- or down-regulated (Fig. 1b,c). The levels of Fru6P;Glc6P were significantly higher (~2.0-fold) in the mutants, and the Glc1P level was consistently somewhat higher (Fig. 1d). Also, the citrate, Gly3P, GlcNAc6P, and S7P levels increased significantly in the mutants (Fig. 1c,d; Table S1). Interestingly, the UDP-Glc level was significantly reduced (up to ~0.6-fold) in all three *fugu5* strains (Fig. 1d; Table S1). Principal component analysis (PCA) of the above metabolic changes indicated that the WT and *fugu5* strains clearly differed (Fig. 1e). On the other hand, the levels of several amino acids were significantly reduced in the *fugu5* strains (Fig. 1c; Table S1). Of the enzymes active on the above metabolites, only UDP-Glc pyrophosphorylase (UGPase) produces PPi. Given that gluconeogenesis is compromised in *fugu5* mutants^13^, the results suggest that UGPase was the likely target of inhibition by excess cytosolic PPi in the *fugu5* background.

**Figure 2:**
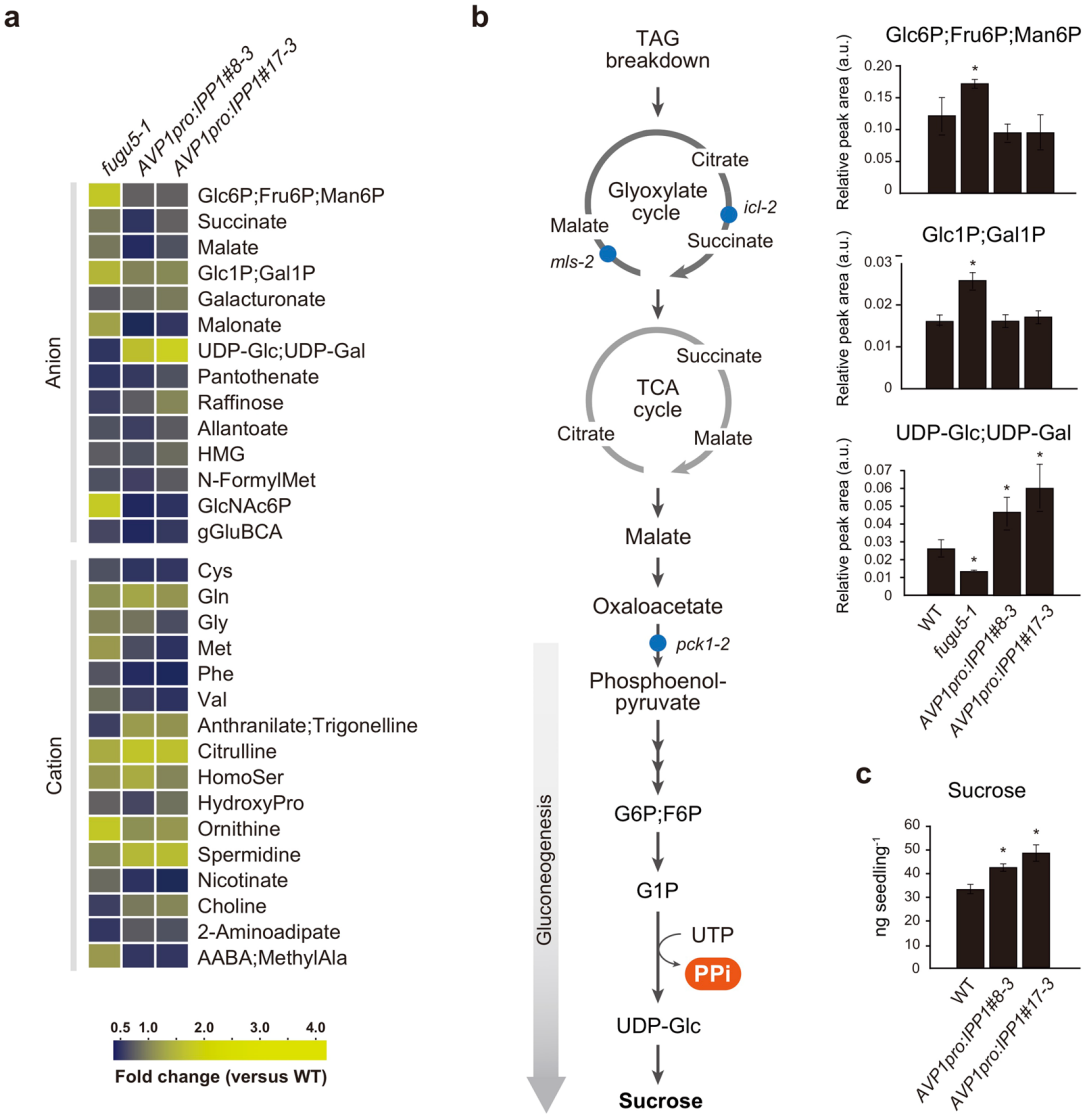
The effect of PPi removal on metabolic fluctuations in the *fugu5* mutant background. (**a**) The levels of certain metabolites in *fugu5*, *AVP1*_*pro*_*:IPP1*#8-3, *AVP1*_*pro*_*:IPP1*#17-3, and the WT strains. Metabolites, the levels of which increased or decreased significantly as revealed by the two-tailed Student’s *t*-test (*P* < 0.05, *n* = 3) are color-coded, and the fold changes in the *fugu5*, *AVP1*_*pro*_*:IPP1*#8-3, and *AVP1*_*pro*_*:IPP1*#17-3 transgenics compared with the WT are shown. Top: anions; bottom: cations. Metabolites that are not resolved by CE-TOF MS (Glc6P, Fru6P, and Man6P; Glc1P and Gal1P; and UDP-Glc and UDP-Gal) are shown together. (**b**) The pathway of sucrose biosynthesis from seed storage lipids during germination and the relative levels of [Glc6P, Fru6P, Man6P], [Glc1P, Gal1P], and [UDP-Glc, UDP-Gal]. Data are means ± SDs (*n* = 3). (**c**) Sucrose concentration in etiolated seedlings of the WT, *AVP1*_*pro*_*:IPP1*#8-3, and *AVP1*_*pro*_*:IPP1*#17-3 transgenics. Data are means ± SDs (*n* = 3). Asterisks indicate significant differences compared to the WT (*P* < 0.05, two-tailed Student’s *t*-test). Abbreviations are summarized in Supplementary Table S3.

To confirm this hypothesis, two *AVP1*_*pro*_*:IPP1* transgenic lines^13^, in which cytosolic PPi is specifically hydrolyzed in the *fugu5* background, were subjected to CE-TOF MS analysis along with WT and a representative *fugu5-1* strain (Fig. 2). The *fugu5* metabolic defects were reversed in *AVP1*_*pro*_*:IPP1* (Fig. 2a), where Glc1P and the Glc6P;Fru6P levels equaled or were only slightly lower than those in WT, respectively (Fig. 2b; Table S2). Additionally, the UDP-Glc levels in *AVP1*_*pro*_*:IPP1*#8-3 and *AVP1*_*pro*_*:IPP1*#17-3 were 1.74- and 2.25-fold higher, respectively, than in the WT (Fig. 2b; Table S2). In other words, simply removing PPi from the *fugu5* background increased the levels of UDP-Glc by 3.4- and 4.5-fold in *AVP1*_*pro*_*:IPP1*#8-3 and *AVP1*_*pro*_*:IPP1*#17-3 respectively (Fig. 2b; Table S2). Finally, to assess the impact of PPi removal on gluconeogenesis, we measured Suc levels. Consistently, the Suc level was 45% higher in *AVP1*_*pro*_*:IPP1*#8-3 and 31% higher in *AVP1*_*pro*_*:IPP1*#17-3 (Fig. 2c). Again, provided that the UGPase-catalyzed reaction is readily reversible^16,22^, our data strongly indicate that UGPase is a major metabolic target of excess PPi *in vivo*.

**Figure 3:**
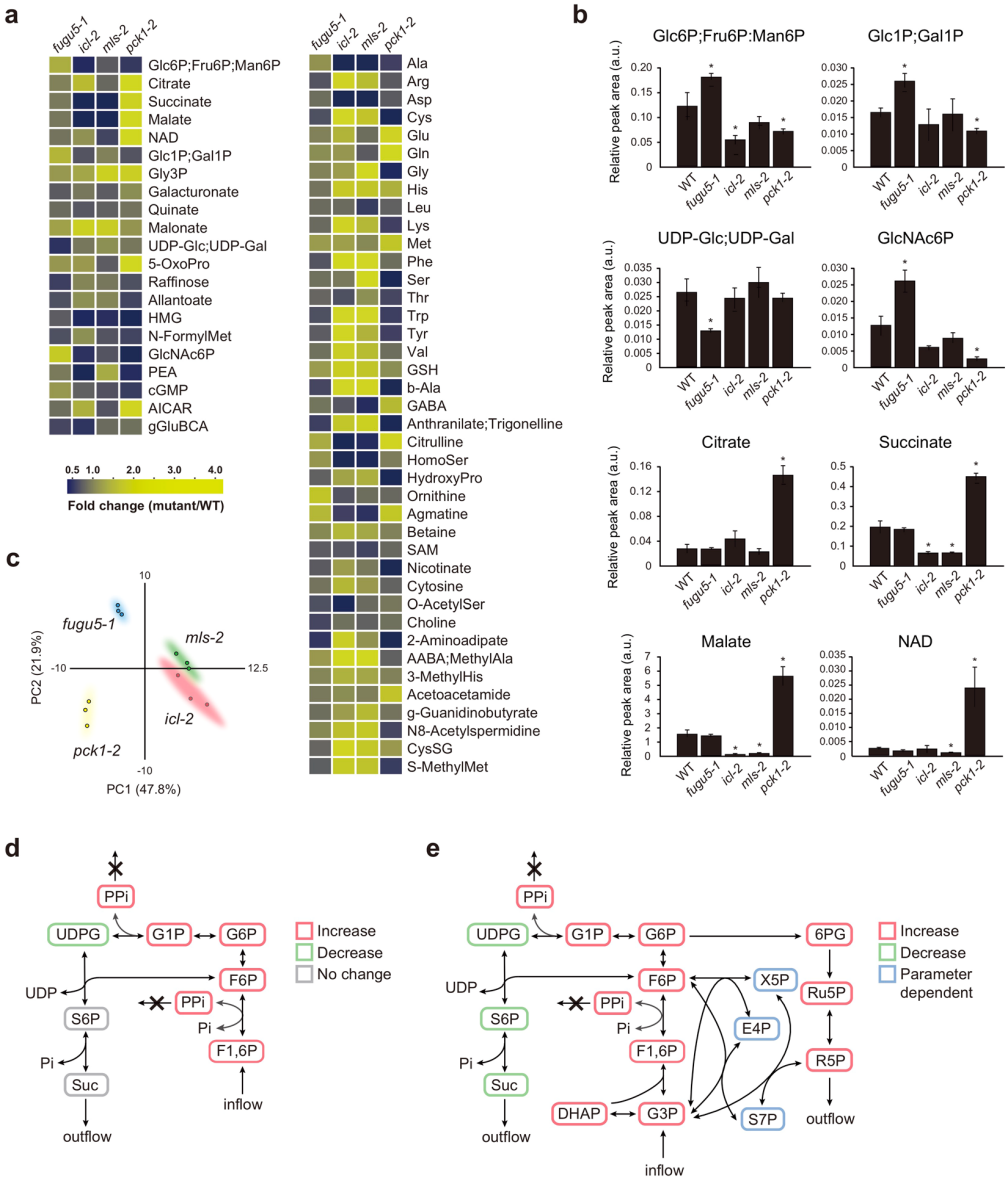
Comparative metabolomics of the *fugu5-1*, *icl-2*, *mls-2*, *pck1-2*, and WT strains and SSA analysis of the metabolic network. (**a**) The levels of certain metabolites in the *fugu5-1*, *icl-2*, *mls-2*, *pck1-2*, and WT strains. Metabolites, the levels of which increased or decreased significantly as revealed by the two-tailed Student’s *t*-test (*P* < 0.05, *n* = 3) are color-coded, and the fold changes in the *fugu5-1*, *icl-2*, *mls-2*, and *pck1-2* strains are compared with those in the WT. Metabolites that are not resolved by CE-TOF MS (Glc6P, Fru6P, and Man6P; Glc1P and Gal1P; and UDP-Glc and UDP-Gal) are shown together. (**b**) Key metabolites, the levels of which were significantly (and differentially) affected in different mutants. Data are means ± SDs (*n* = 3). Asterisks indicate significant differences compared to the WT (*P* < 0.05, two-tailed Student’s *t*-test). (**c**) Score plot of principal component analysis (PCA). (**d**) Structural sensitivity analysis of sucrose production. Perturbation reflects knockout of H^+^-PPase, which mediates PPi degradation. Colors indicate changes in concentrations induced by perturbation in metabolite flow caused by excess PPi. Gray: no change in concentration; green: decrease, red: increase. (**e**) Analysis of the revised network, which explains the observed decreases in S6P and Suc levels. Abbreviations are summarized in Supplementary Table S3.

TAG mobilization is a multistep process that has been extensively investigated; several key enzymes have been identified^23^. The mutants *icl-2*, *mls-2*, and *pck1-2* are defective in isocitrate lyase (ICL), malate synthase (MS), and phopshoenolpyruvate carboxykinase (PEPCK), respectively (Fig. 2b)^24–26^. All exhibit significant reductions in Suc synthesis from TAG and mimic the *fugu5* developmental defects^27^. Therefore, comparative analyses of the metabolic profiles of these three mutants would confirm whether UGPase was a specific target of excess PPi, as the *icl-2*, *mls-2*, and *pck1-2* mutants express the functional H^+^-PPase. Interestingly, CE-TOF MS metabolic profiling revealed that key metabolites, such as Glc6P;Fru6P, Glc1P, and UDP-Glc, were differentially affected in the *icl-2*, *mls-2*, and *pck1-2* strains compared to the *fugu5* strain (Fig. 3a,b). For example, although the UDP-Glc level was significantly reduced in the *fugu5* strain, the levels were almost unaffected in the *icl-2*, *mls-2*, and *pck1-2* strains (Fig. 3b; Table S2). The Glc1P and Glc6P;Fru6P levels were elevated in the *fugu5* strain, but reduced in the *icl-2*, *mls-2*, and *pck1-2* strains (Fig. 3b; Table S2). Additionally, the levels of malate, succinate, and citrate were 3.5-, 2.3-, and 5.4-fold higher, respectively, in the *pck1-2* strain than in WT, indicating that metabolic flow was severely suppressed (Fig. 3b; Table S2). On the other hand, the succinate and malate levels were severely reduced (by up to ~40% and ~10%, respectively, compared to the WT strain) in both the *icl-2* and *mls-2* mutants (Fig. 3b; Table S2). Finally, PCA of the metabolic changes in the three mutants indicated that they all clearly differed (Fig. 3c). Taken together, the results clearly indicated that UDP-Glc production was disrupted by excess PPi *in planta*.

Figure 3d shows the metabolic pathway responsible for Suc production from Fru1,6P_2_. The circuit contains two reversible reactions in which PPi is released as a byproduct. At first glance, any defect in H^+^-PPase would be expected to result in excess PPi in plant cells, reducing the forward rate of reaction, eventually reducing Suc production. We used structural sensitivity analysis (SSA)^28,29^ to analyze the effect of the *fugu5* mutation on metabolic dynamics. The qualitative responses of the steady state metabolite concentrations and fluxes induced by perturbations in enzyme levels can be determined from the network structure alone; it is unnecessary to assume reaction rate functions or parameter values. When applied to the metabolic pathway responsible for Suc production from Fru1,6P_2_ (Fig. 3d), SSA (surprisingly) predicted that excess PPi should not influence the concentration of Suc if the network structure of Fig. 3d was, in fact, correct. This indicated that the network structure had to be revised to explain the experimentally measured metabolite profile of the *fugu5* strains. After analysis of all possible modifications (Fig. S1), we found that addition of at least one of four reactions (involving Fru1,6P_2_, Fru6P, Glc6P, and/or Glc1P) was required to explain the empirical results (please see the Methods for details). Figure 3e shows the structure of one network that successfully explains the observed decrease in Suc levels and also predicts changes in the concentrations of other metabolites caused by excess PPi. Thus, SSA not only allows information on known networks to be validated but also predicts the existence of unsuspected reactions that explain the observed responses of various systems.

PPi is universally produced in large amounts by a variety of vital biosynthetic reactions^5,7^. The UGPase-catalyzed reaction is readily reversible; the *in vivo* equilibrium point depends on the PPi concentration^5,16,22^. Plant cells contain substantial levels of PPi in the cytosol; these remain remarkably constant under a variety of conditions. It has been suggested that PPi-dependent phosphofructokinase (PFP) acts to control PPi levels, but the cytosolic PPi level of transgenic plants with very low levels of this enzyme are barely affected, suggesting that other mechanisms are involved^30^. H^+^-PPase has been proposed as a potential key player in PPi metabolism. However, no *H*^+^*-PPase* mutants were available, and any possible role for the enzyme thus remained unresolved^22^. The widespread belief that proton pumping is the major role of H^+^-PPase is partially supported by the increased tolerance to abiotic stress of many crops engineered to overexpress the enzyme to date^17^, but any possible contribution made by PPi hydrolysis has been overlooked^9,13,18^. Characterization of *fugu5* mutants revealed the pivotal role played by H^+^-PPase in PPi homeostasis and suggested that gluconeogenesis might be compromised by excess PPi^13^. Here, we used a comparative metabolomics approach to confirm that gluconeogenesis is, indeed, affected and provide robust evidence that UGPase is a major target of excess PPi *in vivo*.

UGPase is an important enzyme for the metabolism of UDP-Glc, a key precursor in the synthesis of Suc, cellulose, and callose^15,31,32^, and is thought to be regulated by substrate availability alone at the enzyme level^31^. UGPase has been purified from a wide variety of organisms, including yeast, plants, animals, the slime mold *Dictyostelium discoidium*, and several bacterial species^33^. Although UGPase has been suggested to be classified structurally into both prokaryotic and eukaryotic groups, they have almost identical catalytic properties^34^. The Arabidopsis genome contains three genes encoding UGPase: AtUGP1, AtUGP2, and AtUGP3^35,36^. We have previously demonstrated that recombinant AtUGP3 catalyzed the formation of UDP-Glc from Glc1P and UTP^36^. Moreover, we examined the effect of PPi on the UGPase activity of recombinant UGP3 and found that the addition of various PPi concentrations (0–10 mM) strongly inhibited the UGPase activity of the recombinant UGP3^36^, as has been reported for other UGPases, via product inhibition (see ref.^36^, and the citations therein for details). Thus, the enhanced stress tolerance of crops constitutively expressing H^+^-PPase is, in part, attributable to increased photosynthetic efficiency, and UGPase is a novel useful target in efforts to genetically engineer crops with increased yields^17,18^.

## Methods

### Plant material and growth conditions

Arabidopsis Col-0 was the WT strain, and all the mutants and transgenics were in the Col-0 background. The *fugu5* mutants and the *AVP1*_*pro*_*:IPP1*#8-3 and *AVP1*_*pro*_*:IPP1*#17-3 strains have been previously described^13^. Seeds of the *icl-2*, *mls-2*, and *pck1-2* strains were the kind gift of Professor Ian Graham (the University of York) and have been described previously^24–26^. The seeds were sterilized and sown in plates containing Murashige and Skoog Plant Salt Mixture (Wako Pure Chemical Industries), 0.1% (w/v) 2-(*N*-morpholino) ethanesulfonic acid (MES) (pH = 5.8 as adjusted with KOH), and 0.2% (w/v) gellun gum^37^. The seeds were left in the dark for 3 days at 4 °C, exposed to light (50 µmol m^−2^ s^−1^) to facilitate germination at 22 °C for 6 h, and then maintained at 22 °C in the dark for 66 h. Three sets of experiments were conducted in parallel for each genotype. The first set featured the WT, *fugu5-1*, *fugu5-2*, and *fugu5-3* strains. The second set included the WT, *fugu5-1*, *AVP1*_*pro*_*: IPP1*#8-3, *AVP1*_*pro*_*: IPP1*#17-3, *icl-2*, *mls-2*, and *pck1-2* strains. Three-day-old etiolated seedlings were immediately frozen in liquid nitrogen after sampling and stored at −80 °C prior to CE-TOF MS analysis.

### Metabolite profiling with CE-TOF MS

About 50-mg quantities of frozen seedlings were homogenized using a Zirconia bead in a Safe-seal micro tube (2 mL; PP; Sarsted) with the aid of a Mixer Mill (Retsch). Then, 500 µL methanol containing internal standards (each 8 µM; methionine sulfone and camphor 10-sulfonic acid for cation and anion analysis, respectively) was added, followed by repeat homogenization and centrifugation at 20,400 *g* for 3 min at 4 °C. Next, 500 µL chloroform and 200 µL water were added. The mixture was vortexed for 3 min and centrifuged at 20,400 *g* for 3 min at 4 °C. Methanol in the mixture was evaporated in a centrifugal concentrator for 30 min at 45 °C. The resulting upper layer (100–200 µL water associated fraction) was centrifugally filtered through a Millipore 5-kDa-cutoff filter (Merck Millipore) at 9,100 *g* for 120 min. The filtrate was evaporated to dryness in a centrifugal concentrator for 120 min. The residue was dissolved in 20 µL water and other internal standards (200 µM of each of 3-aminopyrrolidine and trimesic acid for cation and anion analysis, respectively) added before CE-TOF MS analyses. All compounds and solvents were purchased from Sigma-Aldrich (St. Louis, MO) and Wako Pure Chemical Industries (Osaka, Japan), respectively.

CE-TOF MS experiments were performed using an Agilent G7100A CE Instrument (Agilent Technologies), an Agilent G6224A TOF LC/MS, an Agilent G1311C 1260 Infinity Quat Pump VL, a G1603A Agilent CE-MS adapter, an Agilent G1607-60002 CE ESI Sprayer II (Agilent Technologies), and G1601BA Agilent ChemStation Ver. B.04.03 software. Separations were performed with the aid of a capillary filled with fused silica (50 mm internal diameter × 100 cm total length) filled with 1 M formic acid (FA) or 20 mM ammonium formate (pH 10.0) as the electrolyte for cation or anion analyses, respectively. The capillary temperature was maintained at 20 °C. Fifteen nanoliters of sample solution was injected under 50 mbar for 15 s. The sample tray was held at <10 °C. Prior to each run, the capillary was flushed with the electrolyte for 5 min. The voltage used for separation was 30 kV. Methanol/water (50%, v/v) containing 0.5 µM reserpine served as the sheath liquid, delivered at 10 µL/min. ESI-TOF MS was conducted in the positive-ion mode for cation analyses and in the negative-ion mode for anion analyses, and the capillary voltage was set to 4 kV. The flow rate of hot (300 °C) dry nitrogen was 10 psig. The fragmentor, skimmer, and Oct1 RF Vpp values were set to 105, 65, and 750 V, respectively, for cation analysis and to 100, 60, and 750 V, respectively, for anion analysis. Automatic recalibration of each spectrum was performed using the masses of reference standards. The methanol dimer ion ([2 M + H]^+^, *m/z* = 65.0597) and reserpine ion ([M + H]^+^, *m/z* = 609.2806) (cation analyses) or the FA dimer ion ([2M–H]^-^, *m/z* = 91.0037) and reserpine ion ([M–H]^-^, *m/z* = 607.2661) (anion analyses) afforded the lock masses allowing precise mass measurements. Mass data were acquired at 1.5 cycles/s over the 50–1,000 *m/z* range. Further experimental information has been presented elsewhere^38,39^. The raw CE-TOF MS data were converted, and the peaks were automatically identified, aligned, and annotated, using our in-house software (“Masterhands”)^40^. Suc levels in 3-day-old etiolated seedlings were measured as described previously^13^. The two-tailed Student’s *t*-test was used for statistical evaluations. Principle component analysis (PCA) was performed as described previously^41^.

### Structural sensitivity analysis

Structural sensitivity analysis is a mathematical method to determine responses of steady state concentrations and fluxes in chemical reaction networks to the perturbation of each of reaction rate parameters from structure of networks alone^28,29^. In the following, we label chemicals by *m* (*m* = 1, …, *M*) and reactions by *i* (*i* = 1, …, *R*). In general, the state of a chemical reaction system is specified by the concentrations *x*_*m*_ (*t*), which obey the following differential equations

$$\frac{d{x}_{m}}{dt}=\sum _{i=1}^{R}{\nu }_{mi}{W}_{i}({k}_{i};x)$$

Here, the matrix *v* is called a stoichiometric matrix. *W*_*i*_ is called a flux, which depends on metabolite concentrations and also on a reaction rate parameter *k*_*i*_, which corresponds to amount/activity of enzyme mediating the reaction. We do not assume specific forms for the flux functions, but assume that each *W*_*i*_ is an increasing function of its substrate concentration:

$\frac{\partial {W}_{i}}{\partial {x}_{m}} > 0$ if *x*_*m*_ is a substrate of reaction *i*,

$$\frac{\partial {W}_{i}}{\partial {x}_{m}}=0$$ otherwise.

Below, we abbreviate and emphasize nonzero $\frac{\partial {W}_{i}}{\partial {x}_{m}}$ as *r*_*im*_.

In this framework, enzyme knockdown of the *j*th reaction corresponds to changing the reaction coefficient as ${k}_{j}\to {k}_{j}+\delta {k}_{j}$. We assume steady state of this system both before knockdown and after knockdown leading the following condition:

$$\sum _{i=1}^{R}{\nu }_{mi}{\delta }_{j}{W}_{i}=\sum _{i=1}^{R}{\nu }_{mi}(\frac{\partial {W}_{i}}{\partial {k}_{j}}+\sum _{m^{\prime} =1}^{M}\frac{\partial {W}_{i}}{\partial {x}_{m^{\prime} }}\frac{\partial {x}_{m^{\prime} }}{\partial {k}_{j}})\delta {k}_{j}=0$$

Here ${\delta }_{j}\overrightarrow{W}$ is the flux change induced by the parameter change, which is also written as

$${\delta }_{j}\overrightarrow{W}=\sum _{n=1}^{{N}_{k}}{\overrightarrow{c}}^{n}{\delta }_{j}{\mu }^{n}\cdot $$

Here, $\{{\vec{c}}^{1},\ldots ,{\vec{c}}^{{N}_{k}}\}$ is a basis of the right-kernel space of the stoichiometric matrix *v*.

As shown in^28,29^, the metabolite concentration change ${\delta }_{j}\overrightarrow{x}=\frac{\partial \overrightarrow{x}}{\partial {k}_{j}}\delta {k}_{j}$ and flux change ${\delta }_{j}\overrightarrow{W}$ at a steady state under the perturbation ${k}_{j}\to {k}_{j}+\delta {k}_{j}$ are given from network structure only. From a linear algebra derivation, we have a systematic method to determine response of each chemical to perturbation of each reaction rate in a system at the same time:

$$(\frac{{\delta }_{1}\overrightarrow{x}\cdots {\delta }_{R}\overrightarrow{x}}{{\delta }_{1}\overrightarrow{\mu }\cdots {\delta }_{R}\overrightarrow{\mu }})=-\,{{\bf{A}}}^{-1}diag.(\frac{\partial {W}_{1}}{\partial {k}_{1}}\delta {k}_{1},\cdots ,\frac{\partial {W}_{R}}{\partial {k}_{R}}\delta {k}_{R})\equiv {\bf{S}}\,diag.(\frac{\partial {W}_{1}}{\partial {k}_{1}}\delta {k}_{1},\cdots ,\frac{\partial {W}_{R}}{\partial {k}_{R}}\delta {k}_{R}),$$

where the square matrix **A** is given as

$${\bf{A}}=(\frac{\partial {W}_{i}}{\partial {x}_{m}}|-\,{\overrightarrow{c}}^{1},\cdots ,-{\overrightarrow{c}}^{{N}_{k}})\cdot $$

The matrix ${\bf{S}}\equiv -\,{{\bf{A}}}^{-1}\,\,$is called the sensitivity matrix. The flux change is obtained through the following equation

$$({\delta }_{1}\overrightarrow{W}\cdots {\delta }_{R}\overrightarrow{W})=({\overrightarrow{c}}^{1}\cdots {\overrightarrow{c}}^{{N}_{k}})({\delta }_{1}\,\overrightarrow{\eta }\cdots {\delta }_{R}\,\overrightarrow{\eta })$$

Note that distribution of nonzero entries in the matrix **A** reflects structure of reaction network. We determine qualitative response of each chemical and flux from distribution of nonzero entries in the matrix **A** only. This implies that our theory depends only on the structure of reaction network.

The metabolite pathway for Suc production in plant (Fig. 3d) consists of the following 15 reactions:

1. F1,6P → F6P + PPi
2. F6P + PPi → F1,6P
3. F6P → G6P
4. G6P → F6P
5. G6P → G1P
6. G1P → G6P
7. G1P → PPi + UDPG
8. PPi + UDPG → G1P
9. UDPG + F6P → S6P
10. S6P → UDPG + F6P
11. S6P → Suc
12. Suc → S6P
13. PPi → *ϕ* (degradation)
14. Suc → *ϕ* (outflow)
15. *ϕ* → F1,6P (inflow)

The stoichiometry matrix *v* is given by

$$\nu =(\begin{array}{ccccccccccccccc}-\,1 & 1 & 0 & 0 & 0 & 0 & 0 & 0 & 0 & 0 & 0 & 0 & 0 & 0 & 1\\ 1 & -\,1 & -\,1 & 1 & 0 & 0 & 0 & 0 & -\,1 & 1 & 0 & 0 & 0 & 0 & 0\\ 1 & -\,1 & 0 & 0 & 0 & 0 & 1 & -\,1 & 0 & 0 & 0 & 0 & -\,1 & 0 & 0\\ 0 & 0 & 1 & -\,1 & -\,1 & 1 & 0 & 0 & 0 & 0 & 0 & 0 & 0 & 0 & 0\\ 0 & 0 & 0 & 0 & 1 & -\,1 & -\,1 & 1 & 0 & 0 & 0 & 0 & 0 & 0 & 0\\ 0 & 0 & 0 & 0 & 0 & 0 & 1 & -\,1 & -\,1 & 1 & 0 & 0 & 0 & 0 & 0\\ 0 & 0 & 0 & 0 & 0 & 0 & 0 & 0 & 1 & -\,1 & -\,1 & 1 & 0 & 0 & 0\\ 0 & 0 & 0 & 0 & 0 & 0 & 0 & 0 & 0 & 0 & 1 & -\,1 & 0 & -\,1 & 0\end{array}),$$

where the raw indices correspond to {F1,6P; F6P; PPi; G6P; G1P; UDPG; S6P; Suc}.

The matrix **A** is computed as

$${\bf{A}}=(\begin{array}{ccccccccccccccc}{r}_{1,{\rm{F}}1,6{\rm{P}}} & 0 & 0 & 0 & 0 & 0 & 0 & 0 & 2 & 0 & 0 & 0 & 0 & 0 & 1\\ 0 & {r}_{{\rm{2}},{\rm{F6P}}} & {r}_{2,{\rm{PPi}}} & 0 & 0 & 0 & 0 & 0 & 0 & 0 & 0 & 0 & 0 & 0 & 1\\ 0 & {r}_{3,{\rm{F6P}}} & 0 & 0 & 0 & 0 & 0 & 0 & 1 & 0 & 0 & 0 & 0 & 1 & 0\\ 0 & 0 & 0 & {r}_{4,{\rm{G}}6{\rm{P}}} & 0 & 0 & 0 & 0 & 0 & 0 & 0 & 0 & 0 & 1 & 0\\ 0 & 0 & 0 & {r}_{5,{\rm{G}}6{\rm{P}}} & 0 & 0 & 0 & 0 & 1 & 0 & 0 & 0 & 1 & 0 & 0\\ 0 & 0 & 0 & 0 & {r}_{6,{\rm{G}}1{\rm{P}}} & 0 & 0 & 0 & 0 & 0 & 0 & 0 & 1 & 0 & 0\\ 0 & 0 & 0 & 0 & {r}_{7,{\rm{G}}1{\rm{P}}} & 0 & 0 & 0 & 1 & 0 & 0 & 1 & 0 & 0 & 0\\ 0 & 0 & {r}_{8,{\rm{PPi}}} & 0 & 0 & {r}_{8,{\rm{UDPG}}} & 0 & 0 & 0 & 0 & 0 & 1 & 0 & 0 & 0\\ 0 & {r}_{9,{\rm{F6P}}} & 0 & 0 & 0 & {r}_{9,{\rm{UDPG}}} & 0 & 0 & 1 & 0 & 1 & 0 & 0 & 0 & 0\\ 0 & 0 & 0 & 0 & 0 & 0 & {r}_{10,{\rm{S}}6{\rm{P}}} & 0 & 0 & 0 & 1 & 0 & 0 & 0 & 0\\ 0 & 0 & 0 & 0 & 0 & 0 & {r}_{11,{\rm{S}}6{\rm{P}}} & 0 & 1 & 1 & 0 & 0 & 0 & 0 & 0\\ 0 & 0 & 0 & 0 & 0 & 0 & 0 & {r}_{12,{\rm{Suc}}} & 0 & 1 & 0 & 0 & 0 & 0 & 0\\ 0 & 0 & {r}_{13,{\rm{PPi}}} & 0 & 0 & 0 & 0 & 0 & 3 & 0 & 0 & 0 & 0 & 0 & 0\\ 0 & 0 & 0 & 0 & 0 & 0 & 0 & {r}_{14,{\rm{Suc}}} & 1 & 0 & 0 & 0 & 0 & 0 & 0\\ 0 & 0 & 0 & 0 & 0 & 0 & 0 & 0 & 2 & 0 & 0 & 0 & 0 & 0 & 0\end{array})$$

By inverting the matrix **A**, the signs of the entries of **S** are determined as

$${\bf{S}}=(\begin{array}{ccccccccccccccc}- & + & - & + & - & + & - & + & - & + & - & + & - & - & +\\ 0 & 0 & - & + & - & + & - & + & - & + & - & + & - & - & +\\ 0 & 0 & 0 & 0 & 0 & 0 & 0 & 0 & 0 & 0 & 0 & 0 & - & 0 & +\\ 0 & 0 & + & - & - & + & - & + & - & + & - & + & - & - & \pm \\ 0 & 0 & + & - & + & - & - & + & - & + & - & + & - & - & \pm \\ 0 & 0 & + & - & + & - & + & - & - & + & - & + & + & - & \pm \\ 0 & 0 & 0 & 0 & 0 & 0 & 0 & 0 & 0 & 0 & - & + & 0 & - & +\\ 0 & 0 & 0 & 0 & 0 & 0 & 0 & 0 & 0 & 0 & 0 & 0 & 0 & - & +\\ 0 & 0 & 0 & 0 & 0 & 0 & 0 & 0 & 0 & 0 & 0 & 0 & 0 & 0 & -\\ 0 & 0 & 0 & 0 & 0 & 0 & 0 & 0 & 0 & 0 & 0 & - & 0 & + & -\\ 0 & 0 & 0 & 0 & 0 & 0 & 0 & 0 & 0 & - & + & - & 0 & + & -\\ 0 & 0 & - & + & - & + & - & - & + & - & + & - & + & + & \pm \\ 0 & 0 & - & + & - & - & + & - & + & - & + & - & + & + & \pm \\ 0 & 0 & - & - & + & - & + & - & + & - & + & - & + & + & \pm \\ 0 & - & + & - & + & - & + & - & + & - & + & - & + & + & -\end{array}),$$

where +, − represent qualitative responses to perturbations. The symbol ± means that the sign depends on quantitative values of *r*_*im*_. The disruption of H^+^-PPase corresponds to the perturbation ${k}_{13}\to {k}_{13}+\delta {k}_{13}$ with $\,\delta {k}_{13} < 0$. The response presented in Fig. 3d is obtained by reversing the signs of the 13th column of the matrix **S**.

## Electronic supplementary material

Supplementary Information

Supplementary Table 1

Supplementary Table 2

Supplementary Table 3

## Data Availability

All data generated or analysed during this study are included in this published article (and its Supplementary Information files).
