# Author Correction: Pyrophosphate inhibits gluconeogenesis by restricting UDP-glucose formation *in vivo*

**DOI:** 10.1038/s41598-019-43447-5

**Published:** 2019-05-21

**Authors:** Ali Ferjani, Kensuke Kawade, Mariko Asaoka, Akira Oikawa, Takashi Okada, Atsushi Mochizuki, Masayoshi Maeshima, Masami Yokota Hirai, Kazuki Saito, Hirokazu Tsukaya

**Affiliations:** 1 Department of Biology, Tokyo Gakugei University, Koganei, Tokyo 184-8501 Japan; 2 Okazaki Institute for Integrative Bioscience, Okazaki, Aichi 444-8787 Japan; 3 National Institute for Basic Biology, Okazaki, Aichi 444-8585 Japan; 4 Department of Basic Biology, School of Life Science, Graduate University for Advanced Studies, Okazaki, Aichi 444-8585 Japan; 5 RIKEN Center for Sustainable Resource Science, Yokohama, 230-0045 Japan; 6 Laboratory of Cell Dynamics, Graduate School of Bioagricultural Sciences, Nagoya University, Nagoya, 464-8601 Japan; 7 Faculty of Agriculture, Yamagata University, Tsuruoka, 997-8555 Japan; 8 Theoretical Biology Laboratory, RIKEN, Wako, 351-0198 Japan; 9 Graduate School of Pharmaceutical Sciences, Chiba University, Chiba, 263-8522 Japan; 10 Department of Biological Sciences, Graduate School of Science, The University of Tokyo, Tokyo, 113-0033 Japan; 11 Present Address: Exploratory Research Center on Life and Living Systems (ExCELLS), Okazaki, Aichi 444-8787 Japan; 12 Present Address: Department of Biology, Tokyo Gakugei University, Nukui-Kita 4-1-1, Koganei, Tokyo 184-8501 Japan

Correction to: *Scientific Reports* 10.1038/s41598-018-32894-1, published online 02 October 2018

In the original version of this Article, Hirokazu Tsukaya was incorrectly affiliated with ‘Department of Biology, Tokyo Gakugei University, Nukui-Kita 4-1-1, Koganei, Tokyo, 184-8501, Japan’. The correct affiliations are listed below.

Okazaki Institute for Integrative Bioscience, Okazaki, Aichi, 444-8787, Japan

Department of Biological Sciences, Graduate School of Science, The University of Tokyo, Tokyo, 113-0033, Japan

Present address: Exploratory Research Center on Life and Living Systems (ExCELLS), Okazaki, Aichi, 444-8787, Japan

This error has now been corrected in the PDF and HTML versions of the Article.

