# Supplementary material for: Pyrophosphate inhibits gluconeogenesis by restricting UDP-glucose formation in vivo
Source: Sci Rep. 2018 Oct 2;8:14696. doi: 10.1038/s41598-018-32894-1 (PMC6168488; doi:10.1038/s41598-018-32894-1)
Supplement: Supplementary file 1 — Supplementary Information [file 41598_2018_32894_MOESM1_ESM.pdf]

Supplementary Information

Pyrophosphate inhibits gluconeogenesis by restricting UDP-glucose formation *in vivo*

Ali Ferjani[1,*], Kensuke Kawade[2,3,4,5,11], Mariko Asaoka[6,12], Akira Oikawa[5,7], Takashi Okada[8], Atsushi Mochizuki[8,*], Masayoshi Maeshima[6], Masami Yokota Hirai[5], Kazuki Saito[5,9] and Hirokazu Tsukaya[2,10, 11]

[1]Department of Biology, Tokyo Gakugei University, Koganei, Tokyo 184-8501, Japan. [2]Okazaki Institute for Integrative Bioscience, Okazaki, Aichi 444-8787, Japan. [3]National Institute for Basic Biology, Okazaki, Aichi 444-8585, Japan. [4]Department of Basic Biology, School of Life Science, Graduate University for Advanced Studies, Okazaki, Aichi 444-8585, Japan. [5]RIKEN Center for Sustainable Resource Science, Yokohama 230-0045, Japan. [6]Laboratory of Cell Dynamics, Graduate School of Bioagricultural Sciences, Nagoya University, Nagoya 464-8601, Japan. [7]Faculty of Agriculture, Yamagata University, Tsuruoka 997-8555, Japan. [8]Theoretical Biology Laboratory, RIKEN, Wako 351-0198, Japan. [9]Graduate School of Pharmaceutical Sciences, Chiba University, Chiba 263-8522, Japan. [10]Department of Biological Sciences, Graduate School of Science, The University of Tokyo, Tokyo 113-0033, Japan. [11]Present address: Exploratory Research Center on Life and Living Systems (ExCELLS), Okazaki, Aichi 444-8787, Japan.[12]Present address: Department of Biology, Tokyo Gakugei University, Nukui-Kita 4-1-1, Koganei, Tokyo 184-8501, Japan.

Supplemental Methods

Law of localization

The law of localization, which is the direct result from structural sensitivity analysis, determines the extent to which a perturbation influences a network. For a given network, we consider a pair $\Gamma = (\mathfrak{m}, \mathfrak{r})$ of a metabolite subset $\mathfrak{m}$ and a reaction subset $\mathfrak{r}$ satisfying the condition that $\mathfrak{r}$ includes all reactions influenced by metabolites in m. The choice of $\mathfrak{r}$ for a chosen $\mathfrak{m}$ is not unique in general. We call a subnetwork satisfying this condition “output-complete.” For such a subnetwork Γ, we count the number $|\mathfrak{m}|$ of elements in $\mathfrak{m}$, the number $|\mathfrak{r}|$ of elements in $\mathfrak{r}$, and the number $N_k(\mathfrak{r})$ of the closed cycles that consist of the reaction subset $\mathfrak{r}$. Then, we compute an index,

$$\lambda(\Gamma) \equiv -|\mathfrak{m}| + |\mathfrak{r}| - N_k(\mathfrak{r})$$

which is analogous to the Euler characteristic and generally non-negative. The law of localization states that if $\lambda(\Gamma) = 0$ for an output-complete subnetwork Γ, then any perturbation of reactions in Γ does not change the concentrations and the fluxes outside of Γ, namely, the perturbation effect is localized in Γ itself. We call an output-complete subnetwork satisfying $\lambda(\Gamma) = 0$ a "buffering structure."

The original network shown in Fig. 3d includes a buffering structure $\Gamma = (\{\mathrm{F1{,}6P, F6P, G6P, G1P, UDPG, PPi}\}, \{1, 2, 3, 4, 5, 6, 7, 8, 9, 10, 13\})$ with $\lambda(\Gamma) = -6 + 11 - 5 = 0$. From the property of buffering structure, we can derive the following conclusion directly. (1) Any perturbations (not only the disruption of H^+-PPase, but also other reaction coefficients)

in the reactions in buffering structures should not change concentrations in S6P and Suc, because these two molecules are outside of the buffering structure.

(2) an additional reaction from/to any metabolites within the buffering structure will not change the condition of the buffering structure, because such modification will increase both reaction number and cycle number, namely $\lambda(\Gamma') = -6 + 12 - 6 = 0$.

(3) the condition of buffering structure is broken by adding reactions from five metabolites (F1,6P, F6P, G6P, G1P, UDPG) in the buffering structure to any metabolites outside of the structure.

By the structural sensitivity analysis shown above, the signs of sucrose response to perturbation of H^+-PPase are determined. By adding reactions from the four metabolites (F1,6P, F6P, G6P, G1P, UDPG), the sucrose concentration will decrease by the knockdown of H^+-PPase. On the other hand, by adding a reaction from UDP-Glc, the sucrose concentration will increase by the knockdown of H^+-PPase. From these results, we predict existence of reactions from either of F1,6P, F6P, G6P, G1P, UDPG. The summary of the knockdown influence to the concentration of Suc in the presence of these additional reactions is shown in Fig. S1.

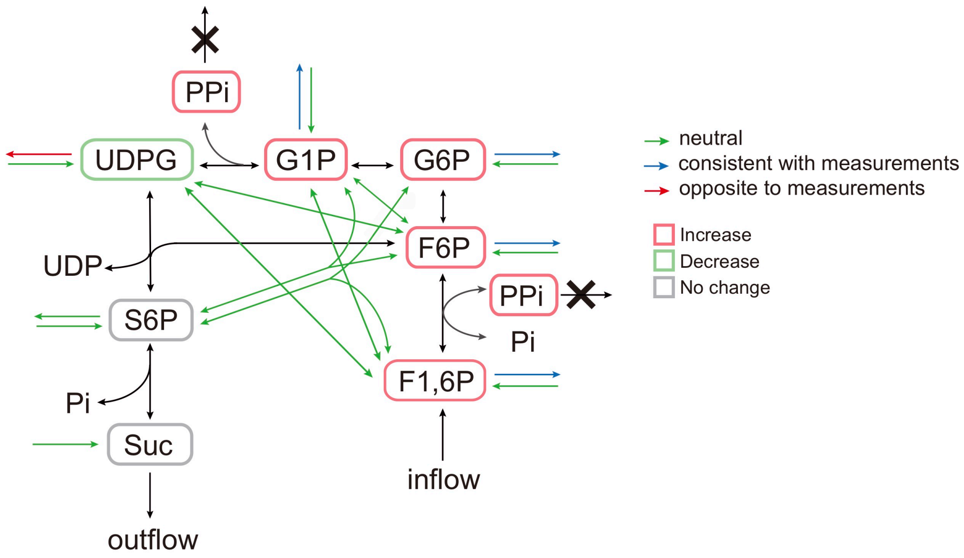

Supplementary Figure 1. Summary of modifications to the basic network.

Colors indicate changes in concentrations induced by perturbation in metabolite flow caused by excess PPi. Gray: no change in concentration; green: decrease, red: increase. Abbreviations are summarized in Supplementary Table S3.
